# Diffusion weighted magnetic resonance imaging (DW-MRI) as a non-invasive, tissue cellularity marker to monitor cancer treatment response

**DOI:** 10.1186/s12885-020-6617-x

**Published:** 2020-02-19

**Authors:** Frederikke Petrine Fliedner, Trine Bjørnbo Engel, Henrik H. El-Ali, Anders Elias Hansen, Andreas Kjaer

**Affiliations:** 1 Department of Clinical Physiology, Nuclear Medicine & PET and Cluster for Molecular Imaging, Department of Biomedical Sciences, Rigshospitalet and University of Copenhagen, Copenhagen, Denmark; 2 Department of Biomedical Sciences, Rigshospitalet and University of Copenhagen, Copenhagen, Denmark; 3 Colloids and Biological Interface Group, Department of Micro- and Nanotechnology, Technical University of Denmark, Lyngby, Denmark; 4 Section of Cellular and Metabolic Research, Department of Biomedical Sciences, University of Copenhagen, Copenhagen, Denmark

**Keywords:** Diffusion weighted MRI, Cancer treatment response, ADC-value, Preclinical, Cellular density

## Abstract

**Background:**

Diffusion weighted magnetic resonance imaging (DW-MRI) holds great potential for monitoring treatment response in cancer patients shortly after initiation of radiotherapy. It is hypothesized that a decrease in cellular density of irradiated cancerous tissue will lead to an increase in quantitative apparent diffusion coefficient (ADC) values. DW-MRI can therefore serve as a non-invasive marker of cell death and apoptosis in response to treatment. In the present study, we aimed to investigate the applicability of DW-MRI in preclinical models to monitor radiation-induced treatment response. In addition, we compared DW-MRI with ex vivo measures of cell density, cell death and apoptosis.

**Methods:**

DW-MRI was tested in two different syngeneic mouse models, a colorectal cancer (CT26) and a breast cancer (4 T1). ADC values were compared with quantitative determinations of apoptosis and cell death by flow cytometry. Furthermore, ADC-values were also compared to histological measurement of cell density on tumor sections.

**Results:**

We found a significant correlation between ADC-values and apoptotic state in the CT26 model (*P* = 0.0031). A strong correlation between the two measurements of ADC-value and apoptotic state was found in both models, which were also present when comparing ADC-values to cell densities.

**Conclusions:**

Our findings demonstrate that DW-MRI can be used for non-invasive monitoring of radiation-induced changes in cell state during cancer therapy. ADC values reflect ex vivo cell density and correlates well with apoptotic state, and can hereby be described as a marker for the cell state after therapy and used as a non-invasive response marker.

## Background

Monitoring of treatment response in cancer patients is of huge clinical importance to optimize therapeutic interventions, and the general approach based on morphology is described by the RECIST guidelines which were last updated in 2009 [1]. However, non-invasive measures of functional changes in the tumor, e.g. induction of cell death and cell density, may be of complementary value for response monitoring.

One such potential imaging modality is diffusion weighted magnetic resonance imaging (DW-MRI). Initially, this technique was focused on neuroimaging due to the limited motion in the brain and hereby a decreased number of pitfalls and artifact shortcomings [2]. However, an increased methodical knowledge and less hardware limitations has led to the use of DW-MRI in most parts of the body [3].

DW-MRI is based on the brownian motion within tissues, and molecule-movement being restricted by cellular structures in high-density tissue e.g. solid cancers [4–6]. In more detail, DW-MRI measures the indirect value of cellularity by applying the same gradient at continuous short time intervals. The movement of water molecules causes loss of signal through spin dephasing, and an apparent diffusion coefficient (ADC) value can be defined from the signal loss over time [7, 8]. A high ADC-value hereby represents a steep slope of signal loss and vice versa. It has been shown that there is an inverse correlation between cellular density and ADC, describing a high cellular density as a low ADC-value due to high restriction in tissue and hereby decreased water movement [9–11].

DW-MRI is described as a promising way to non-invasively monitor treatment response shortly after treatment initiation. Several clinical and preclinical studies are currently ongoing or published on the use DW-MRI as a prognostic marker in various cancers [3, 6, 9, 12–18]. Accordingly, it has been shown that in general there is an increase in ADC-value following effective treatment. However, although the majority of studies find an increase in ADC-value following therapy and a correlation with long term survival or disease progression, contradictory and conflicting results have also been reported [19, 20]. Furthermore, recommendations for the use of DW-MRI was discussed in 2008 during “The International Society for Magnetic Resonance in Medicine Meeting” held in Toronto. Concerns for the lack of understanding DW-MRI at a microscopic level was among the points to be summarized in the meeting report [21].

The aim of this study was therefore to evaluate the feasibility of DW-MRI treatment response monitoring of external radiotherapy and to evaluate how DW-MRI correlates with changes in cell density and induction of apoptosis in a preclinical setting to obtain knowledge of the robustness of the method for translational purposes.

To do so, we studied the correlation between the non-invasively collected ADC-values in tumors before and after irradiation and ex vivo measures of cell density and cell apoptosis by immunohistochemistry and flow cytometry in two different murine models.

## Methods

### Tumor model

All experimental procedures were approved by the Danish Animal Welfare Council, the Danish Ministry of Justice (license no. 2016-15-0201-00920). Mice were housed in IVC rack in Type III SPF cages with a maximum of 8 mice in each cage. Food and water was available ad libitum at all times.

Tumors were grown on female BalbC mice (Charles River, Scanbur A/S, Karlslunde, Denmark) and mice were included at 8 weeks of age after 1 week of acclimatization. Mice were injected subcutaneously with either 3 × 10^5^ CT26 WT (murine colon carcinoma, CRL-2638, ATCC, Virginia, USA) cells or 5 × 10^5^ 4 T1 (murine stage IV breast cancer cells, CRL-2539, ATCC, Virginia, USA) in a total volume of 100 μL RPMI serum-free medium on the lower part of the right flank. Roswell Park Memorial Institute (RPMI) medium supplemented with 10% Fetal Calf Serum (FCS) and 1% penicillin-streptomycin (Biowest, Nuaillé, France) was used for growth of both cell lines prior to inoculation. In vitro growth of cells upon inoculation were maintained in culture flasks (5% CO_2_ at 37^0^ C). During inoculation mice were anaesthetized with 3.5% sevoflurane (Abbvie Inc., North Chicago, IL, USA) in a mixture of oxygen and air (35% O_2_ and 65% N_2_). Tumor size and body weight were measured continuously from day 5 after implant to follow the development of tumors and monitor health of the mice. Tumor size were calculated from the formula of 0.52 · (W^2^ · L), where L represents the length and W for the width measured by external caliper. When tumors reached a mean size of approximately 170 mm^3^, mice were randomized into three different groups of six mice for both models. Groups included an untreated control group and two treatment groups receiving either 10 Gy or 15 Gy of radiation therapy. Motivations for treatment doses were chosen based on the known sensitivity of the included tumor models towards radiation therapy to investigate doses inducing a treatment response, but without diminishing investigated tumors completely during study time in order to sustain tumor tissue for sampling at day 4. Filatenkov and colleagues have shown how 30 Gy leads to complete remission in CT26 tumors and the doses were set based on this and studies within our own department showing treatment responses from doses of 10 and 15 Gy in both models [22]. Radiation therapy was delivered at 1 Gy/min (320 kV/12.5 mA) using a biological irradiator X-Rad 320 (PXI Precision X-ray, North Branford, Connecticut, USA).

### MRI protocol

DW-MRI scans were performed using a Preclinical BioSpec MR 7 T Scanner (Bruker, Ettlingen, Germany) and a 20 mm planar RF surface coil. Both an anatomical T2-weighted sequence and diffusion-weighted sequence was acquired. The T2-weighted anatomical sequence was performed using the following parameters; TR/TE. 2500/35 milliseconds, image size: 256 × 256, Field of view (FOV): 30 × 30 mm, averages: 2, slice thickness: 0.7 mm, and scan time 2 min 40 s. Diffusion-weighted EPI scan sequence was performed using the following parameters; TR/TE: 550/24 milliseconds, image size: 96 × 96, FOV: 30 × 30 mm, averages: 6, segments: 6, slice thickness: 0.7 mm, b-values: 0, 100, 200, 600, 1000, 1500, 2000, and scan time 2 min 18 s. An extended shimming procedure and B_0_-map was included in diffusion-weighted protocol to decrease artifacts and noise in images.

All mice had MRI performed at baseline before radiation therapy and daily for 4 days following irradiation to monitor treatment response. Mice were anesthetized as previously described, placed on a water-heated bed to stabilize body temperature, and respiration was monitored during entire scan procedure.

Image analysis was performed in ParaVision 6.0.1 software (Bruker, Ettlingen, Germany). Region of interests (ROI) were drawn over tumor tissue in a circular shape on a single axial slice placed to cover center of tumor in a maximum volume. Tissue ADC-values were calculated using bi-exponential signal intensity plot fitting, and results accordingly describe the mean ADC-value in a central slice of the tumor.

### Apoptosis quantification by Annexin V

After MRI scan on day four after radiation therapy, all mice were euthanized by cervical dislocation and tumors collected for ex vivo analysis. Tumors were harvested and stored in MACS tissue storage solution until dissociated with Tumor Dissociation kit (Miltenyi Biotec, Germany) using a gentleMACS™ Octo Dissociator (Miltenyi Biotec, Germany). Dissociation was performed following the manufacturer’s standard protocol. Cells were washed and diluted into single cell suspensions before assay detection, and red blood cells lysed using VersaLyse™ Lysing Solution, according to manufacturer’s protocol (Beckman Coulter, Brea, Californien, USA). Detection of apoptotic cells, apoptotic stage, and dead cells in tumor tissue was obtained using MUSE® Cell Analyzer and accompanying Annexin V & Dead Cell Kit (Merck Millipore, Darmstadt, Germany) [23].

Results of the cellular counts are given in percentages of the gated cells and in number of total cells counted in the gated area. Gates were set in a default setting on a test sample of tissue, and kept fixed for all samples in data set. Results are presented as percentage of all apoptotic cells, including both cells gated as early apoptotic and late apoptotic.

### Immunohistochemistry protocol

Immunohistochemistry (IHC) was performed on formalin-fixed, paraffin-embedded 4 μm tumor sections that were stained with haematoxylin and eosin for 5 and 3 min, respectively. Separate groups of mice were included for this study. A total of 4 mice were included for each treatment group in the two different models of either CT26 or 4 T1. Subsequently slides were mounted for electronic slide scanning (Axio scan, Carl Zeiss, Germany) (pixel size 0.022 × 0.022 μm). Five regions of interests were used to generate a reflection of the full slide environment. Cellular density was determined using the “color deconvolution”-function in Fiji [24] to isolate the haematoxylin-stain image. The isolated haematoxylin stained image was extracted to binary values and the nuclei density was determined by excluding fragments and artifacts by automated exclusion of structures below a cut-off size of 50 pixels^2^. All structures above 50 pixels^2^ were hereby counted as cellular nuclei, and the “watershed”-function was used to differentiate if stacks of nuclei were seen, and hereby counted as individual nuclei. Five density values are thereby included for each tumor slide, and two individual slides for each tumor was evaluated.

### Statistical analysis

Statistical analysis was performed in GraphPad 7 (GraphPad Software, San Diego, CA, USA). Results of tumor volumes are presented as mean ± SEM (Standard Error of Mean). Analysis of data from tumor growth, ADC-values, histological staining, and apoptotic assay was performed using one-way ANOVA variance analysis to evaluate differences over time or between treatment groups. Pearson correlation analysis was used to evaluate correlations between data sets. *P*-value <0.05 was considered statistically significant in all cases.

## Results

DW-MRI scan sequence was performed with high qualitative reproducibility and limited artifacts. Optimal image quality seen in center of coil, but no eddy currents- or motion artifacts were seen in either of the slices, which is otherwise known to be an obstacle for EPI sequences.

**Fig. 1:**
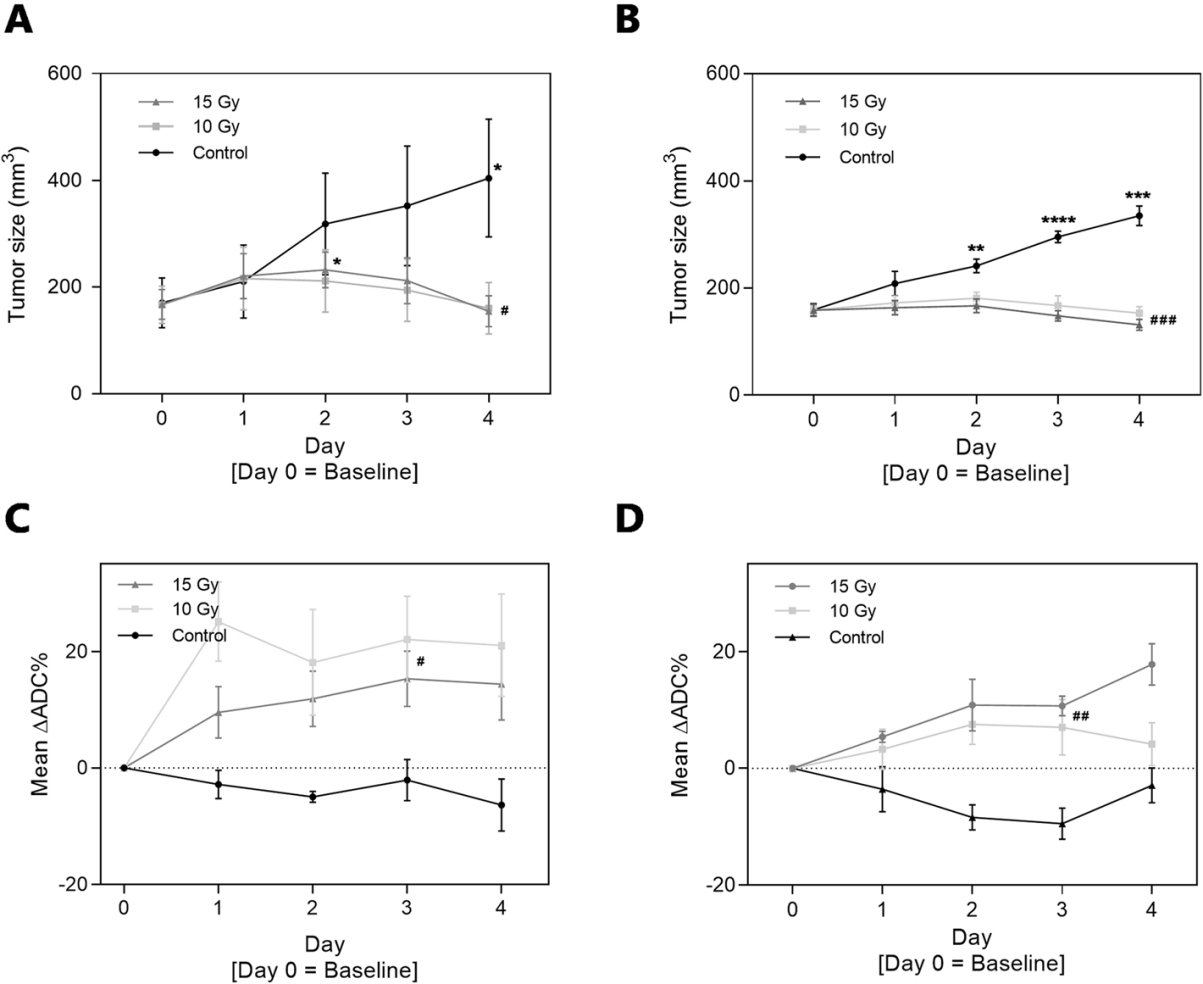
Tumor volumes (mm^3^) calculated from external caliper measurements for tumor-bearing mice of both CT26 (**a**) and 4 T1 model (**b**). Volumes are described by mean ± SEM (*n* = 6 mice/group). Irradiation was performed on Day 0. Bottom row presents systematic ADC-results over time in percent compared to baseline (mean ± SEM) for CT26 (**c**) and 4 T1 tumors (**d**), respectively. ADC-values are extracted from MRI scans in ROI volume including central part of tumor. *) *p* < 0.05; **) *p* < 0.01, ***) *p* < 0.001: represents the systematic changes in tumor size for each group over time (one-way ANOVA calculations on repeated measurements), and the #) *p* < 0.05; ##) *p* < 0.01, ###) *p* < 0.001 represents differentiation between groups from one-way ANOVA at marked day in figure, e.g. tumor size at Day 4 comparison

Tumor growth for all groups in both models are shown in Fig. 1a and b as mean ± SEM in each group. Successful tumor inhibition was seen in all treatment groups for both models over time given by a significant increase in control groups, which was absent in all treatment groups. The average non-treated CT26 tumor size (± SEM) increased 240% from 170 ± 47 mm^3^ to 400 ± 110 mm^3^ from day 0 to day 4. In the group treated with 10 Gy, a decrease in size of 7% from 170 ± 35 mm^3^ to 160 ± 48 mm^3^ was observed, and in the 15 Gy treated group a decrease of 9% in size from 170 ± 28 mm^3^ to 155 ± 29 mm^3^. The tumor size at day 4 of the treated groups were approximately 60% lower than in the untreated group (one-way ANOVA, *P* = 0.0416). Non-treated 4 T1 tumors increased 210% in group mean size (± SEM) from 160 ± 12 mm^3^ to 335 ± 18 mm^3^ from day 0 to day 4. In the group treated with 10 Gy, a mean tumor size decrease of 4% from 160 ± 7 mm^3^ to 153 ± 13 mm^3^ was observed, and in the 15 Gy treated group a tumor size decrease of 19% from 160 ± 11 mm^3^ to 130 ± 10 mm^3^ was observed. The size at day 4 of the treated groups were approximately 55% lower for the 10 Gy group and 60% lower for the 15 Gy group, than in the untreated group (one-way ANOVA, *P* = 0.0001). Successful tumor inhibition is seen in all treatment groups for both models over time given by a significant increase in tumor size for the control groups, which is absent in all treatment groups (significance levels shown in Fig. 1a and b). Larger variations in tumor size were seen for the syngeneic CT26 colon cancer model compared to the syngeneic 4 T1 breast cancer model, which is also depicted by the *p*-values for comparison on day 4.

ADC-values on the DW-MRI scans made on day 0, 1, 2, 3, and 4 increased for treatment groups compared to control group for both models (Fig. 1c and d). ADC-values were defined as the mean value for the center of tumor volume, detected as a circle-shaped ROI to include as much tumor as possible in the chosen slice. ADC results are presented in relative values to describe the systematical changes in tissue after treatment. There was a mean increase of between 15 to 20% for the two treatment groups in the CT26 model compared to a mean decrease of approximately 2–5% in the control group at day 3 (one-way ANOVA, *P* = 0.0190). Equivalent pattern is seen for the 4 T1 model, where a mean increase of approximately 10% in ADC-value for the two treatment groups are seen compared to a 10% decrease in ADC-value for the control group at day 3 (one-way ANOVA, *P* = 0.0014).

**Fig. 2:**
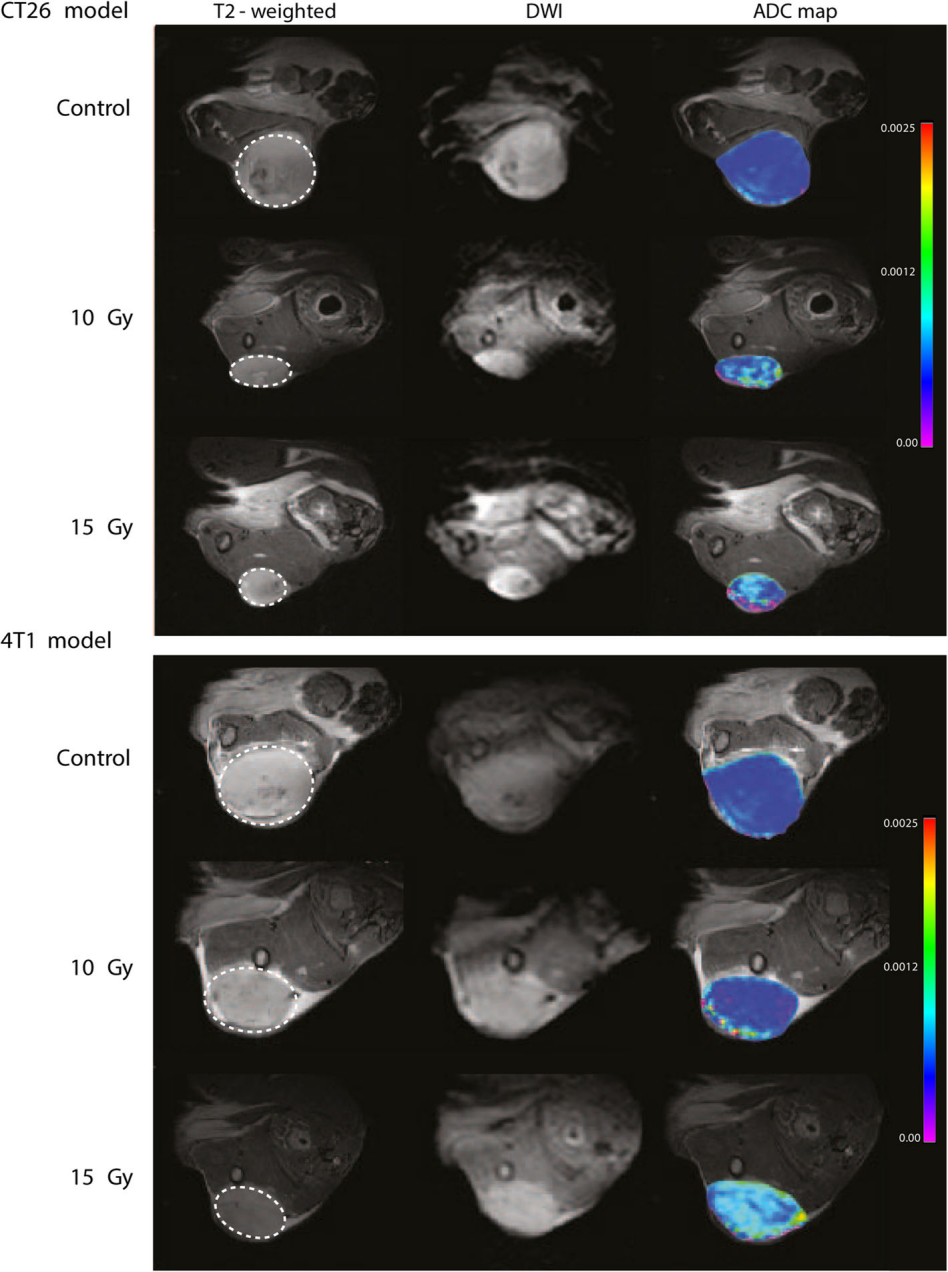
MRI images shown for both anatomical T2-weighted scan, DWI scan from shortest b-value, and overlay of anatomical image and ADC-map. Depicted is one mouse from each group in both models. The T2-weighted anatomical sequence was performed on Bruker 7 T preclinical MRI system using the following parameters; TR/TE. 2500/35 milliseconds, image size: 256 × 256, Field of view (FOV): 30 × 30 mm, averages: 2, slice thickness: 0.7 mm, and scan time 2 min 40 s. Diffusion-weighted scan sequence was performed using the following parameters; TR/TE: 550/24 milliseconds, image size: 96 × 96, FOV: 30 × 30 mm, averages: 6, segments: 6, slice thickness: 0.7 mm, b-values: 0, 100, 200, 600, 1000, 1500, 2000, and scan time 2 min 18 s

Representative examples of MR scans (T2-weighted anatomical scan, DWI and ADC-map) from each group in both models are shown in Fig. 2.

**Fig. 3:**
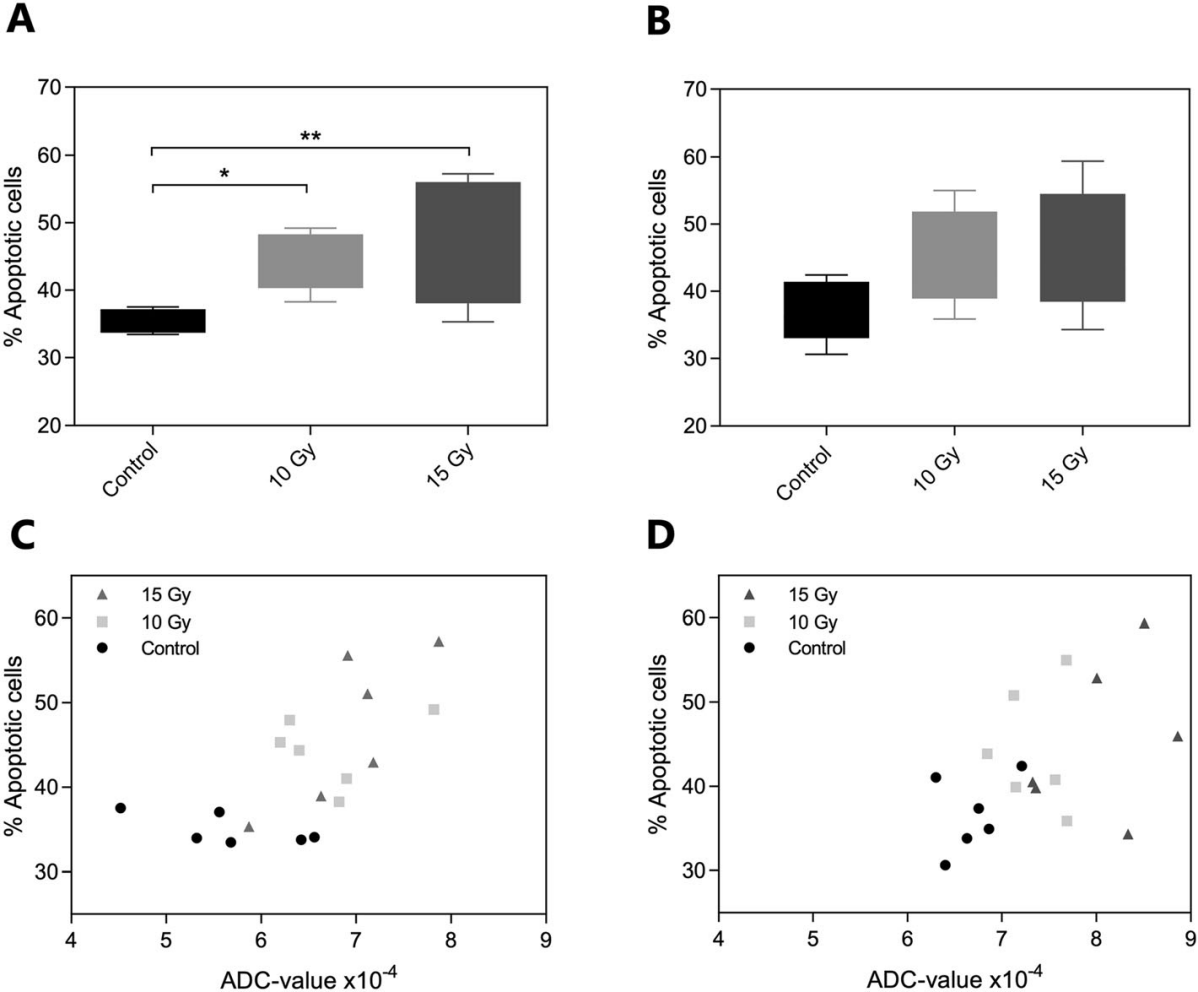
Results of apoptotic cell percentages for tumor-bearing mice of both CT26 (**a**) and 4 T1 (**b**). Results obtained using MUSE® Cell Analyzer and apoptotic cell percentages are described as mean ± SEM in the three treatment groups including six mice each on Day 4 after treatment. Statistical significance was found using one-way ANOVA testing with *p*-value of 0.0077 for CT26 model, but no significance found for 4 T1 model. Bottom row presents correlation of ADC-results at Day 4 and apoptotic cell percentages on the same day for CT26 (**c**) and 4 T1 (**d**), respectively. ADC-values are extracted from MRI scans in ROI volume including central part of tumor, and here presented in original values at Day 4. Correlation analysis was made using Pearson correlation, resulting in values of r = 0.657 and *p* = 0.0031 for the CT26 tumors, and r = 0.508, *p* = 0.0319 for the 4 T1 tumors

Percentages of apoptotic cells determined by ex vivo analysis using Annexin V staining are presented in Fig. 3a and b. Here, a significant increase in apoptotic cell percentages of tumors after treatment compared to untreated tumors was observed in the CT26 model (one-way ANOVA, *p* = 0.0077), presented by an increased apoptotic cell proportion changing from a mean of 35 ± 1% in the control group to a mean of 45 ± 3% in the two treatment groups. Contrarily, no significant differences were found in the 4 T1 model although a similar tendency was apparent, and an increase in apoptotic cell percentages was observed ranging from a mean percentage of 37 ± 2% in the control group to an increase of 45 ± 4% in both treatment groups. Lack of significance is most likely due to increase in variance in the control group for the apoptotic results of the 4 T1 model compared to the CT26 model.

Figure 3c and d illustrates the ADC-values at day 4 for individual tumors compared to the percentage of apoptotic cells from the same tumor. A strong significant positive correlation was found between ADC-values of tumors and corresponding percentage of apoptotic cells for both the CT26 tumors (Pearson r = 0.657, *P* = 0.0031) and the 4 T1 tumors (Pearson r = 0.508, *P* = 0.031).

**Fig. 4:**
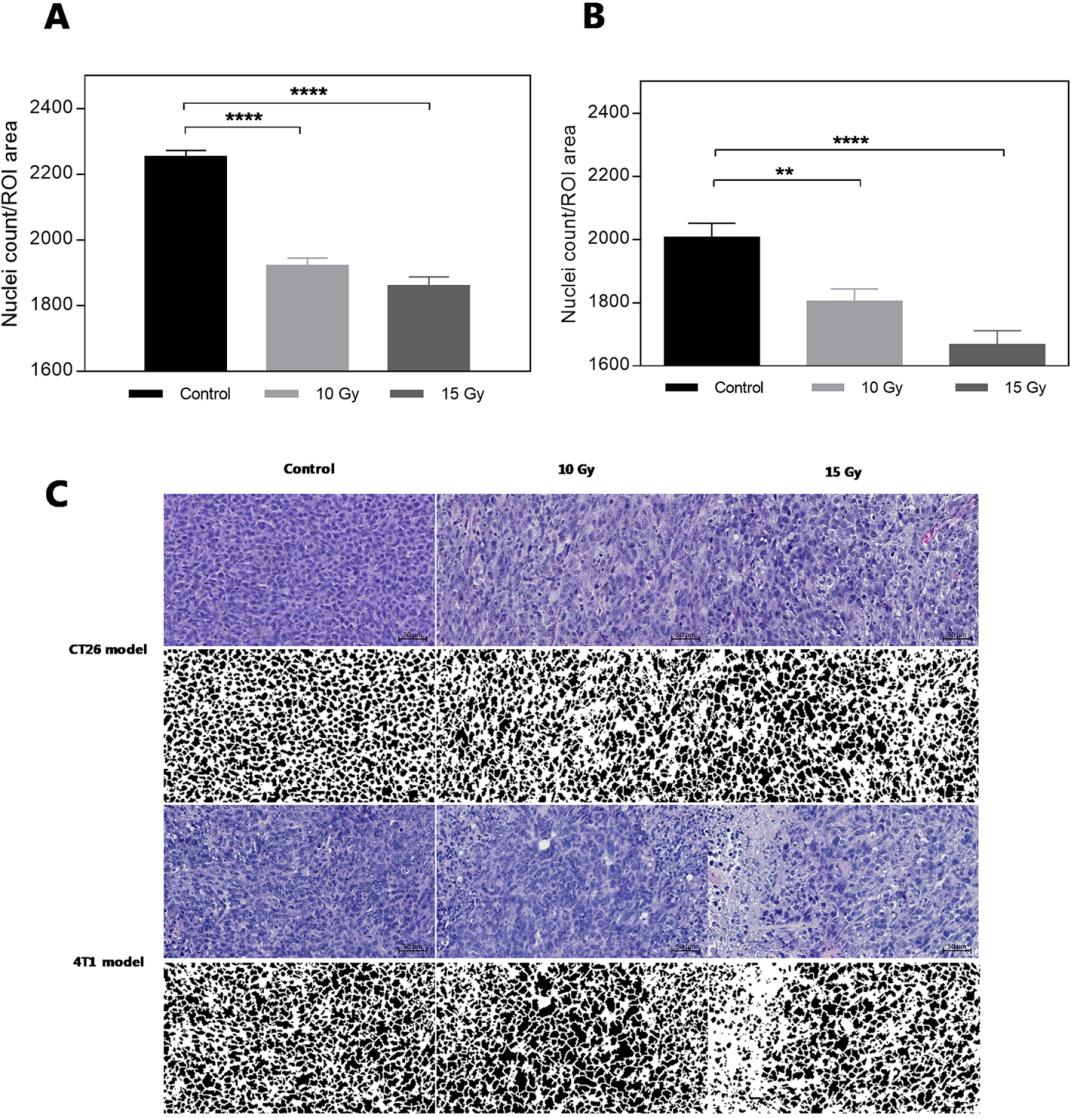
Nuclei count for tumor-bearing mice of both CT26 (**a**) and 4 T1 (**b**) (mean ± SEM, *n* = 8 slides/group). Statistical significance is found using one-way ANOVA testing (*p* < 0.0001 for both models). * depicting statistical differences of multiple comparisons compared to control group. Bottom row (**c**) presents examples from HE staining with paired deconvoluted binary images to show the nuclei count in the two models for all groups. Nuclei counts are calculated as number of nuclei per ROI area from five different ROIs in tumor slide in all groups. The depicted binary images are only for visualization and not does not resemble the ROI used for data analysis

For IHC analysis, additional tumors were collected 4 days after identical radiation treatment and the direct tumor cell-densities were measured by nuclei-staining and counting. Results of tumor-cell nuclei-staining in both tumor models are shown in Fig. 4a and b. Figure 4c illustrate a representative IHC section and an image of the quantification of cell nuclei. From these analyses, a significant decrease in cellular density after radiation treatment is observed compared to untreated controls in both cases (one-way ANOVA, *P* < 0.0001). For the CT26 model a mean group decrease from 2250 ± 18 nuclei per ROI in the control group to 1920 ± 22 nuclei per ROI for the 10 Gy treatment group, and a further mean decrease to 1860 ± 25 nuclei per ROI in the 15 Gy treatment group. Similarly for the 4 T1 model, mean nuclei count per ROI in the control group was 2000 ± 45, and a decrease in group mean for the 10 Gy treatment group results in count of 1800 ± 35 nuclei per ROI compared to a mean of 1660 ± 44 nuclei per ROI for the 15 Gy treatment group.

## Discussion

DW-MRI scans may provide a method of non-invasive measurement of therapeutic efficacy. This is based on the hypothesis that DW-MRI describes an indirect state of cell density in tumor tissue where a lowering in cell density equals an increase in ADC-value [5]. Cell density, size, macromolecules, and myelin layers are among structures that restrict the water movements, which is basis for the proposed hypothesis of using DWI-MRI. DW-MRI is currently being tested in both preclinical and clinical studies [5, 11, 15–17, 25].

In the current study, we found that radiation treatment of tumors increases ADC-values following the induction of cellular damage and apoptosis in the irradiated region, which is in accordance with Baskar et al., stating that apoptosis is one of two main factors leading to cell deatch after irradiation [26]. The validity of the ADC-value as an indirect measure of cellular density was investigated by comparing with ex vivo measurement of cellular density. The ex vivo quantification of cellular density is a direct measurement, and introduces the need for a biopsy of tissue in a clinical setting and is therefore heavily influenced by micro regional differences, e.g. necrotic or hypoxic regions, and thereby sampling error in clinical patients. DW-MRI scans on the other hand can cover larger regions and produce an overall quantitative measurement of important tumor characteristics using a non-invasive and repeatable method. This is very important as clinical tumors are highly heterogeneous in terms of tumor microenvironment and response. To optimally perform the comparisons, tumor tissues for post-mortem analysis were harvested in an un-paired setting, but with identical treatment groups of both included models. Compatible results were found between ex vivo cellular density and ADC values, validating DW-MRI as a non-invasive method for cellular density measurement. Given the high predictability and reproducibility in the two investigated models, it was assumed that tissue state in an un-paired setting corresponds to the tumor samples used for the paired correlation between DW-MRI and apoptotic cell percentages. However, the limitation of no fingerprint comparison between cellular densities and ADC-values needs to be stated when reviewing the results.

The apoptotic cell percentages found in study showed an increase for all treatment groups, but also a rather high percentage in the control groups (approx. 35% apoptotic cells). The level of apoptosis in the control groups could be present due to necrotic regions occurring as a result of increasing tumor size, compared to treated tumors, leading to outgrowth of capacity for neovascularization and formation of hypoxic areas. The heterogeneous structure of the included models is more compatible to clinical tumors, and an advantage of the syngeneic tumor model making them highly resemble to the clinical situation [27, 28]. However, one limitation to be emphasized is that the DW-MRI data origins from on single slice in tumor whereas the apoptotic cell percentages are calculated from whole tumor volume. Spatial correlation between the two parameters are hereby precluded, which could affect the obtained results.

Despite these observations, the DW-MRI method does seem to possess some degree of uncertainty given that the results are highly sensitive and thereby susceptible to heterogeneity in tumor tissue as described above. This may explain the conflicting reporting’s in published studies [29–31]. To use DW-MRI to determine patient outcomes in a clinical setting, a standardized protocol for result validation is mandatory [32]. Yet, this does not exist. A proposed method could be to set a value of percent increase in ADC-value normalized to baseline scan to stratify responders from non-responders at a given time after treatment initiation. Threshold value does, however, need to account for method variance and tissue heterogeneity. For the two different cancer models investigated in our study, a cancer-type specific cellular density was found. The CT26 tumors had higher nuclei count density, and consequently lower ADC-values, compared to the 4 T1 model (data presented in Fig. 3c and d). In addition, the CT26 model seemed more sensitive to irradiation and displayed a more rapid decrease in cellular density and increase in ADC values compared to the 4 T1 model.

A study similar to ours from Paevangelou et al. previously showed that ADC-value can be used as biomarker for early treatment response to cytotoxic drugs [33]. This matches with data obtained in the present study where ADC-values correlated to the cellular density, but not consistently to level of apoptotic cells. In our study the total level of apoptotic cells (including both early and late apoptotic state) correlated to ADC-values. In order for DW-MRI to be used as prognostic marker, results indicate that late cell-death and potentially changes in tissue structure needs to be present at the time of imaging. The use of DW-MRI has been proposed as an individual marker for treatment response [34], but also as a complementary marker to FDG-PET [35].The combination of the non-invasive evaluation of both cellular density (ADC) and metabolic activity could be used to better predict therapeutic outcome, but further clinical studies are needed to verify this.

Studies in different types of cancer have also proposed DW-MRI as a marker to distinguish benign and malignant tumors and to evaluate the aggressiveness of cancer based on ADC-values, but that goes beyond the scope of the present study [36–40].

## Conclusion

Our study found that DW-MRI may be used for response monitoring in radiation therapy. ADC-values reflect both cellular density and apoptosis in the two different tumor types investigated in our study.

## Data Availability

The datasets used and analyzed during the current study are available from the corresponding author on reasonable request.

## Abbreviations

ADC: Apparent diffusion coefficient
DW-MRI: Diffusion weighted magnetic resonance imaging
FOV: Field of view
IHC: Immunohistochemistry
ROI: Region of interests
SEM: Standard Error of Mean
